# CD40L Deficiency Attenuates Diet-Induced Adipose Tissue Inflammation by Impairing Immune Cell Accumulation and Production of Pathogenic IgG-Antibodies

**DOI:** 10.1371/journal.pone.0033026

**Published:** 2012-03-08

**Authors:** Dennis Wolf, Felix Jehle, Alexandra Ortiz Rodriguez, Bianca Dufner, Natalie Hoppe, Christian Colberg, Andrey Lozhkin, Nicole Bassler, Benjamin Rupprecht, Ansgar Wiedemann, Ingo Hilgendorf, Peter Stachon, Florian Willecke, Mark Febbraio, Christoph J. Binder, Christoph Bode, Andreas Zirlik, Karlheinz Peter

**Affiliations:** 1 Atherogenesis Research Group, Department of Cardiology, University of Freiburg, Freiburg, Germany; 2 Baker IDI Heart and Diabetes Institute, Melbourne, Australia; 3 Department of Laboratory Medicine, Medical University of Vienna and Center for Molecular Medicine, Austrian Academy of Sciences, Vienna, Austria; University of Bremen, Germany

## Abstract

**Background:**

Adipose tissue inflammation fuels the metabolic syndrome. We recently reported that CD40L – an established marker and mediator of cardiovascular disease – induces inflammatory cytokine production in adipose cells *in vitro*. Here, we tested the hypothesis that CD40L deficiency modulates adipose tissue inflammation *in vivo*.

**Methodology/Principal Findings:**

WT or CD40L^−/−^ mice consumed a high fat diet (HFD) for 20 weeks. Inflammatory cell recruitment was impaired in mice lacking CD40L as shown by a decrease of adipose tissue macrophages, B-cells, and an increase in protective T-regulatory cells. Mechanistically, CD40L-deficient mice expressed significantly lower levels of the pro-inflammatory chemokine MCP-1 both, locally in adipose tissue and systemically in plasma. Moreover, levels of pro-inflammatory IgG-antibodies against oxidized lipids were reduced in CD40L^−/−^ mice. Also, circulating low-density lipoproteins and insulin levels were lower in CD40L^−/−^ mice. However, CD40L^−/−^ mice consuming HFD were not protected from the onset of diet-induced obesity (DIO), insulin resistance, and hepatic steatosis, suggesting that CD40L selectively limits the inflammatory features of diet-induced obesity rather than its metabolic phenotype. Interestingly, CD40L^−/−^ mice consuming a low fat diet (LFD) showed both, a favorable inflammatory and metabolic phenotype characterized by diminished weight gain, improved insulin tolerance, and attenuated plasma adipokine levels.

**Conclusion:**

We present the novel finding that CD40L deficiency limits adipose tissue inflammation *in vivo*. These findings identify CD40L as a potential mediator at the interface of cardiovascular and metabolic disease.

## Introduction

The incidence of the metabolic syndrome (MS) and its subsequent cardiovascular complications are on the rise worldwide [1]. The MS comprises a cluster of risk factors including visceral obesity, insulin resistance, dyslipidemia, hyperglycemia, hypertension, and hepatic steatosis [2], [3]. Recent evidence demonstrates that visceral obesity associates with chronic low-grade inflammation and up-regulation of inflammatory signaling pathways in adipose tissue [4]. In line with this, adipose tissue has been reported to act as an active endocrine and immune organ efficiently secreting various adipokines including the pro-inflammatory cytokines TNFα and IL-6, the chemokine monocyte chemoattractant protein (MCP)-1, and the procoagulant plasminogen activator inhibitor (PAI)-1 [5], [6], [7], [8]. Several reports indicate that inflammatory and immune cells such as T-cells and their subsets, B-cells, macrophages, and mast cells accumulate in adipose tissue during diet-induced obesity (DIO) and orchestrate adipose tissue inflammation [9], [10], [11], [12], [13], [14], [15]. Impaired lipid metabolism and triglyceride storage by adipocytes is a result of adipose tissue inflammation and excess of free fatty acids aggravates adipose tissue dysfunction [16]. Beyond this local effect, inflamed adipose tissue propagates a systemic inflammation predisposing for insulin resistance and cardiovascular pathologies such as atherosclerosis [17], [18]. Although strong evidence identified inflammation as the key modulator of the metabolic syndrome, exact immunological pathways mediating inflammatory cascades in adipose tissue remain poorly understood [19].

CD40L, a member of the Tumor Necrosis Factor (TNF) superfamily, acts as powerful mediator of adaptive and innate immunity [20]. Beyond its function in immunity and co-stimulatory pathways, CD40L plays a crucial role in a variety of chronic inflammatory pathologies including inflammatory bowel disease, multiple sclerosis, and rheumatoid arthritis [21]. Furthermore, CD40L and its downstream signaling adaptors, such as TNF receptor-associated factors (TRAFs), are potent regulators of atherosclerosis and cardiovascular disease [22], [23], [24], [25], [26]. We and others recently demonstrated that plasma levels of sCD40L associate with visceral obesity and adipokines. Moreover, CD40L correlates with traditional cardiovascular risk factors, such as insulin resistance and hypercholesterolemia [27], [28], [29], [30]. On a mechanistic level, our group and others previously demonstrated that CD40L induces inflammatory cytokine expression in adipocytes, adipogenesis, and the expression of the adipose transcription factors C/EBPα, PPARγ, and Sox-9 in Murine 3T3L1-cells and primary human adipocytes *in vitro* [27], [31]. Notably, CD40 receptor was found to be expressed on human pre-adipocytes and adipocytes and gene expression of CD40 in visceral adipose tissue correlated with body mass index. In the light of these data, we hypothesized that CD40L deficiency modulates adipose tissue inflammation *in vivo*.

## Results

### CD40L deficiency reduces weight gain on low fat diet, but does not prevent the onset of obesity on high fat diet

**Figure 1:**
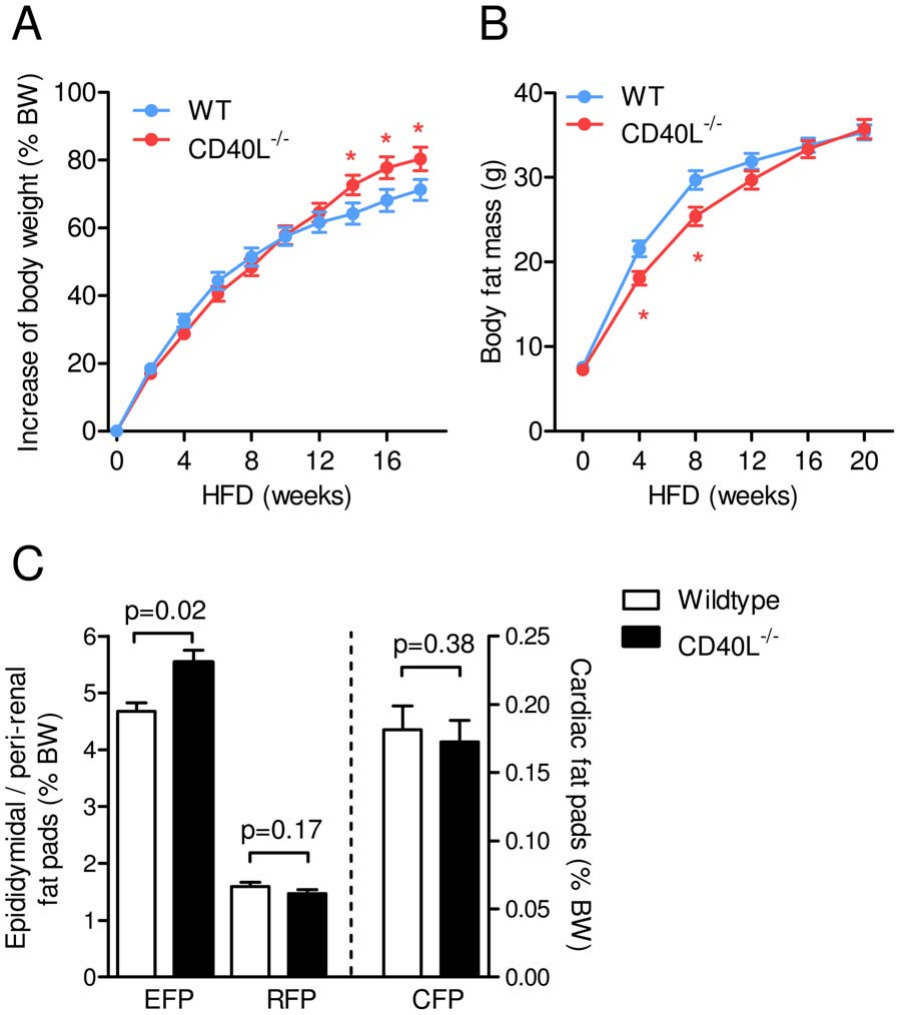
Genetic deficiency of CD40L does not protect from diet-induced obesity. WT and CD40L^−/−^ mice consumed a high fat diet (HFD) for 20 weeks. Relative increase of body weight (shown as % of body weight at week 0) and total body fat as assessed by MRI-based body composition analysis are shown for the indicated time points (A, B). Weight of epididymidal (EFP), peri-renal (RFP), and cardiac fat pads (CFP) fat are displayed as percentage of total body weight (BW, C). Data are presented as mean ± SEM of at least 15 mice per group.

To determine the role of CD40L in diet-induced obesity (DIO) wild type (WT) and CD40L^−/−^ mice consumed either low fat (LFD) or high fat diet (HFD) for 20 weeks. CD40L^−/−^ mice consuming LFD for 20 weeks gained significantly less weight than respective wild type controls throughout the study, resulting in a reduction of body weight gain by 23±8% (p = 0.006, n≥15 per group, Figure S1 A). Accordingly, after 20 weeks on LFD relative body fat of CD40L^−/−^ mice was decreased by 29±7% as assessed by MRI-based body composition analysis (p = 0.0004, n≥15 per group, Figure S1 B). On HFD, absence of CD40L did not affect body weight gain during the first 12 weeks of the study, but significantly increased weight gain at the late stages of DIO by up to 12±6% (p = 0.04, n = 35 per group, Figure 1A). Relative body fat content was slightly reduced during early stages but did not differ at the end of the study (Figure 1 B).

**Figure 2:**
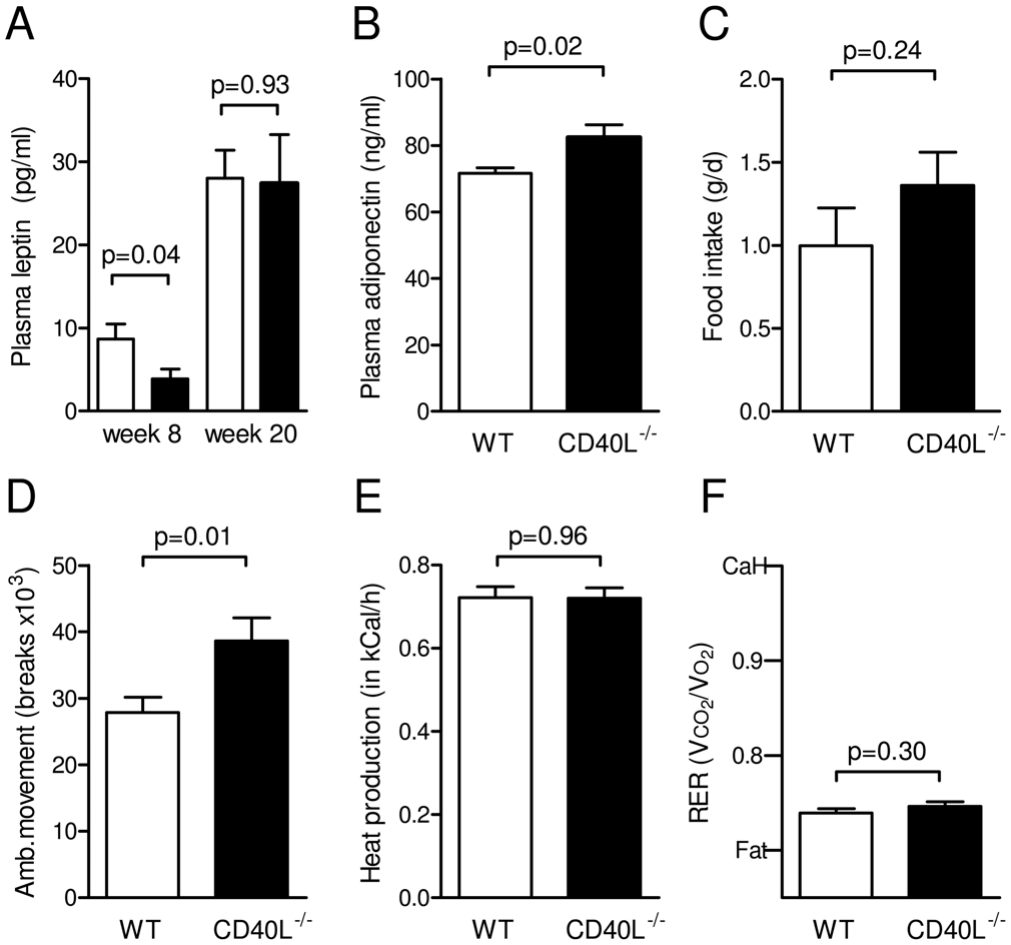
CD40L deficiency does not affect basic energy metabolism. WT and CD40L^−/−^ mice consumed a high fat diet (HFD) for 20 weeks. Plasma levels of leptin (A) and adiponectin (B) were determined at the indicated time points by ELISA. Food intake (C), heat production (D), ambulatory movement (E), and respiratory exchange ratio (RER, F) were analyzed in metabolic cages. CaH and fat indicate carbohydrate and fat metabolism (F). Data are presented as mean ± SEM of at least 9 mice per group.

In line with these data, CD40L^−/−^ mice consuming LFD for 20 weeks contained significantly less peri-renal fat than respective wild-type controls (Figure S1 C), while CD40L^−/−^ mice consuming HFD for 20 weeks contained similar or modestly increased epididymidal fat (Fig. 1 C). Accordingly, plasma leptin levels decreased in the HFD group at week 8 of DIO, but did not significantly differ at the end of the study (Figure 2 A), while adiponectin increased in CD40L-deficient mice after HFD (Figure 2 B). On LFD, plasma leptin was reduced at both time points (Figure S1 D). These effects on DIO could not be attributed to changes in energy metabolism since plasma food intake and heat production assessed by metabolic caging did not differ between both genotypes under both dietary regimens (Figure 2 C–D, Figure S1 E–G). Interestingly, ambulatory movement was even slightly increased in CD40L^−/−^ mice consuming HFD (by up to 39±13%, p = 0.01, n≥20 per group, Figure 2E). Respiratory exchange ratio (RER) was not significantly altered in both groups independently of the diet consumed (Figure 2 F, Figure 1 H). As expected, RER was overall lower in HFD-groups with no detectable difference between WT and CD40L^−/−^ mice. Growth hormone secretion is blunted in obesity [32] and might account for differences in growth and weight. Levels of somatostatin decreased, while levels of growth hormone releasing hormone (GnRH) and Insulin-like growth factor (IGF-1) increased in CD40L-deficient mice consuming LFD (Figure S1 I–K), suggesting a role for CD40L in growth hormone regulation. However, differences in growth hormone levels were not observed in respective mice on HFD.

### CD40L deficiency does not improve high fat diet-induced insulin resistance

**Figure 3:**
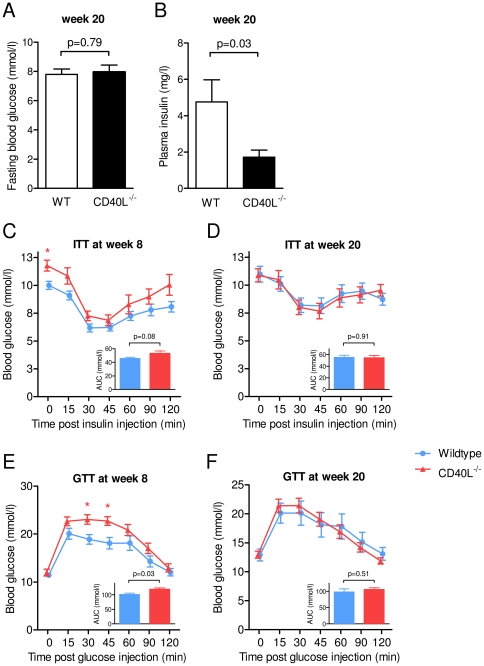
Absence of CD40L does not ameliorate insulin sensitivity in diet-induced obesity. Plasma glucose (A) and insulin levels (B) were determined in animals fasted overnight (A) and for 6 hours (B) overnight. Insulin tolerance (ITT) and glucose tolerance testing (GTT) were performed after intraperitoneal insulin (0.5 U/kg lean body mass) or glucose (1 g/kg lean body mass) injection (C–F). Inlays represent area under the curve calculation (AUC) of the indicated glucose curve.

Insulin resistance and elevated fasting blood glucose levels have been reported to be associated with elevated CD40L plasma levels in humans [28]. As expected, plasma glucose levels were significantly higher in HFD-consuming animals after 8 and 20 weeks of diet compared with animals consuming LFD in our study (data not shown). While glucose levels did not differ between CD40L^−/−^ and wild-type mice at week 20 (Figure 3 B), plasma insulin levels decreased by 64±8% (p = 0.03, n≥5 per group) in CD40L^−/−^ mice consuming HFD (Figure 3 C). These data suggested an increased insulin sensitivity of CD40L-deficient mice. However, CD40L^−/−^ mice did not show improved insulin tolerance after 8 and 20 weeks on HFD compared with corresponding control animals (Figure 3 C, D). Also, CD40L-deficiency did not alter intraperitoneal glucose tolerance testing at both time points of the study (Figure 3 E, F).

To gain more insight into the role of CD40L on insulin sensitivity we performed hyperinsulinemic-euglycemic clamps in HFD-consuming animals. Interestingly, glucose infusion rate (GIR) tended to decrease in CD40L^−/−^ mice compared with WT controls, whereas insulin-mediated glucose disappearance (Ins-GDR) was not altered (Figure 2 A, B). Basal glucose disappearance/appearance (basal GDR) tended to increase in CD40L-deficient mice (Figure S2 C). Glucose uptake into peripheral muscle tissue and heart muscle decreased or tended to decrease in CD40L-deficient mice, while glucose uptake into adipose tissue was only modulated in subcutaneous, but not in visceral or brown adipose tissue in CD40L-deficient mice (Figure S2 E), suggesting tissue-specific peripheral insulin resistance in these mice. Moreover, gene expression of insulin-receptor 1 (IRS-1), glucose-transporter 4 (GLUT-4), and gluconeogensis regulating phosphoenolpyruvate carboxykinase (PEPCK) was potently regulated in different tissues in CD40L^−/−^ mice (Figure S2 F–K), further supporting involvement of CD40L in glucose homeostasis.

### CD40L deficiency aggravates diet-induced hepatic steatosis

**Figure 4:**
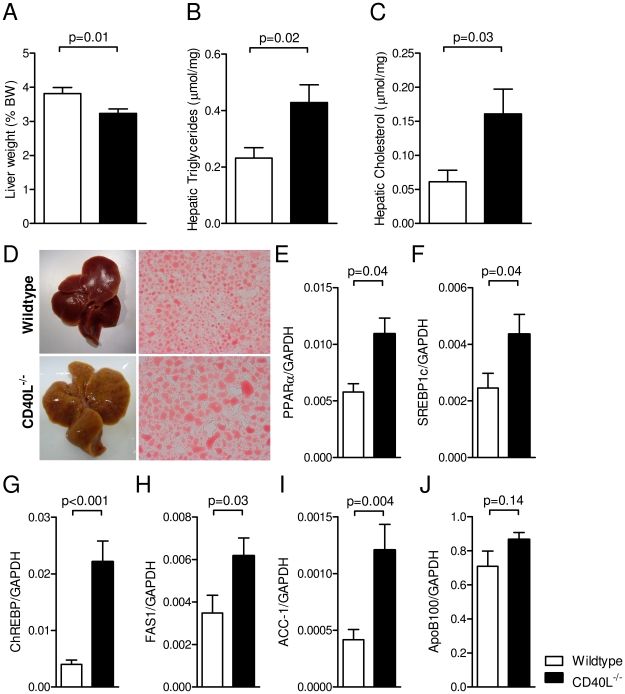
Genetic deficiency of CD40L aggravates diet-induced hepatic steatosis. Liver weight is shown after 20 weeks of HFD (A). Hepatic triglycerides and cholesterol was measured in liver lysates (B,C). Liver sections were stained with lipid-specific oil-red-O (ORO, E). RNA abundance of peroxisome proliferator-activated receptor alpha (PPARα), carbohydrate responsive element-binding protein (ChREBP), sterol regulatory element-binding protein-1 (SREBP1c), free fatty acid synthase (FAS), acetyl-CoA-carboxylase 1 (ACC1), apolipoprotein-B100 (ApoB100) was determined by real-time PCR in whole liver tissue after 20 weeks of HFD (E–J). Data are presented as mean ± SEM of at least 6 animals per group.

Dyslipidemia and non-alcoholic hepatic steatosis (NASH) are well established features of the metabolic syndrome [33]. Villeneuve *et al.* recently reported that CD40L might serve as protective factor in hepatic steatosis – demonstrating progressive hepatic inflammation and hampered secretion of lipoproteins in the blood of CD40L^−/−^ mice [34]. Accordingly, we observed that low-density lipoproteins (LDL) were reduced by 63±8% (p = 0.01, n = 8 per group) in CD40L-deficient mice after 20 weeks of diet, while total plasma cholesterol, triglycerides, and free fatty acids did not differ between CD40L^−/−^ mice and respective controls (Table 1). Interestingly, relative liver weights were reduced in CD40L^−/−^ mice by 15±6% (p = 0.01, n = 22) in the HFD group (Figure 4 A). Hepatic steatosis is characterized by excessive fat accumulation within the liver [35]. Correspondingly, liver lysates from CD40L-deficient mice contained significantly higher amounts of triglycerides regardless of the diet consumed (Figure 4 B). In accord, cholesterol accumulation in the HFD-group increased by 163±60% in CD40L^−/−^ mice (p = 0.03, n = 6, Figure 4 C). Moreover, macroscopic and histological analysis showed enhanced accumulation of fat-specific Oil-red-O-positive staining, augmented lipid droplets, and increased vacuole formation in livers of CD40L−/− mice (Figure 4 D). Accordingly, master regulators involved in lipid metabolism, such as the transcription factors peroxisome proliferator-activated receptor alpha (PPARα), carbohydrate responsive element-binding protein (ChREBP), and sterol regulatory element-binding protein-1 (SREBP1c), as well as factors required for lipid synthesis such as free fatty acid synthase (FAS) and acetyl-CoA-carboxylase 1 (ACC1) were potently regulated in CD40L^−/−^ liver tissue (Figure 4 E–I). However, we did not observe decrease of apolipoprotein-B100 (ApoB100) as previously described [34] (Figure 4 J).

**Table 1:** Baseline study characteristics.

	LFD			HFD		
	WT	CD40L^−/−^	p-value	WT	CD40L^−/−^	p-value
Total body weight at week 0 (g)	25.3±0.4	22.8±0.4	<0.01			
Total body weight at week 20 (g)	41.9±0.7	31.20±0.8	<0.01	42.9±0.6	41.7±0.7	0.18
Lean body mass at week 0 (% body weight)	95.0±0.66	94.1±0.65	0.38	93.9±0.62	93.0±0.50	0.24
Lean body mass at week 20 (% body weight)	76.1±0.7	81.2±1.6	<0.01	64.6±0.7	64.1±0.93	0.69
Heart weight (% BW)	0.41±0.02	0.44±0.01	0.26	0.37±0.02	0.39±0.02	0.41
Spleen weight (% BW)	0.29±0.02	0.21±0.01	<0.01	0.34±0.02	0.29±0.02	0.12
Kidney weight (% BW)	1.1±0.04	1.1±0.03	0.12	1.0±0.04	0.9±0.04	0.69
Blood pressure at week 0 (mmHg)	92.4±1.3	100.1±1.3	<0.01			
Blood pressure at week 20 (mmHg)	94.7±2.7	94.9±2.2	0.94	97.4±1.7	100.7±2.1	0.21
Serum total cholesterol at week 20 (mmol/l)	1.8±0.1	2.0±0.4	0.17	2.7±0.4	2.4±0.4	0.56
Serum LDL-cholesterol at week 20 (mmol/l)	0.12±0.03	0.05±0.01	0.03	0.36±0.06	0.22±0.06	0.1
Serum Triglycerides at week 20 (mmol/l)	0.91±0.09	0.74±0.03	0.1	0.77±0.07	0.74±0.03	0.36
Serum free fatty acids (ng/ml)	ND	ND		449.5±29.9	470.5±21.1	0.57
Adipocyte Cholesterol content/PC	ND	ND		1.25±0.2	1,14±0.3	0.79
CD8^+^ T-cells (% CD3+)	34.9±0.7	30.2±0.8	<0.01	27.8±1.3	29.6±0.6	0.17
CD4^+^ T-cells (% CD3+)	55.8±1.1	60.1±1.2	0.02	56.5±1.2	59.9±0.5	0.01
CD115^+^GR-1^+^ monocytes (%)	18.8±3.6	7.5±1.6	0.02	18.8±2.7	14.9±2.3	0.28
Splenic T-cells (% splenocytes)	28.0±1.0	26.8±0.6	0.37	25.6±0.8	20.9±0.7	<0.01
Splenic T-regulatory cells (%)	12.1±0.5	5.9±0.6	<0.01	13.2±0.4	6.8±0.3	<0.01

Data are presented as mean ± SEM, ND not determined, LFD low fat diet, HFD high fat diet, WT wildtype, PC Phosphatidylcholine, BW body weight.

### CD40L deficiency attenuates diet-induced adipose tissue inflammation and down-regulates local and systemic chemokine gene expression

**Figure 5:**
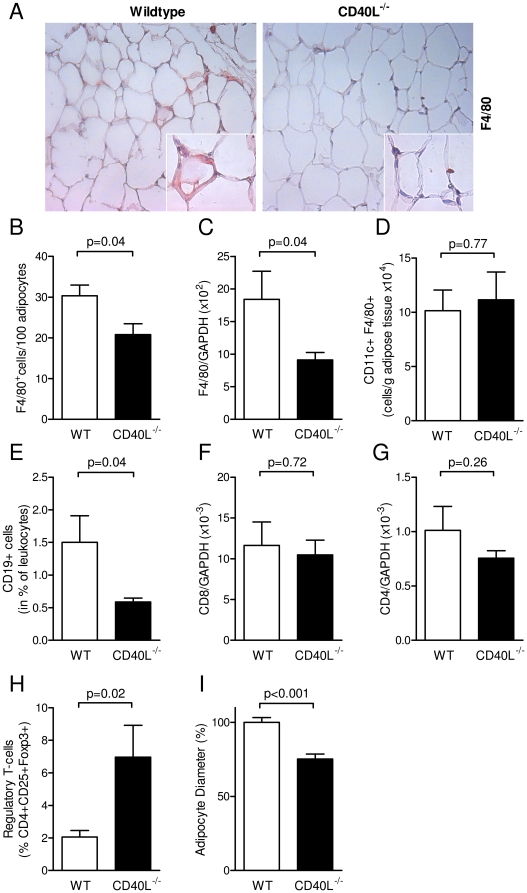
CD40L deficiency attenuates diet-induced adipose tissue inflammation by reduction of immune cell infiltration. Wildtype (WT) and CD40L−/− mice consumed HFD for 20 weeks. Infiltration of macrophages was quantified by detection of the F4/80 antigen in immunohistochemistry (A, B) and quantitative PCR (C). CD11c-expressing M1-macrophages were determined by flow cytometry (D). B-cells were defined as CD19-positive cells in flow cytometry after enzymatic digestion of adipose tissue and specific anti-CD19 staining (C). Numbers of CD8^+^ cytotoxic and CD4^+^ T-helper-cells were quantified by detection of specific mRNA transcripts in whole tissue RNA preparations and normalized to GAPDH (F, G). Regulatory T-cells were defined as CD4^+^CD25^+^FoxP3^+^ cells in flow cytometry (H). Mean adipocyte diameter was calculated in histological sections by image-processing software (I). Data are presented as mean ± SEM of at least 6 animals per group.

Adipose tissue macrophages (ATMs) accumulate in DIO as previously reported and are considered a marker of adipose tissue inflammation [12]. The latter is considered instrumental for the pathogenesis of the metabolic syndrome and its subsequent clinical complications. As expected, macrophage infiltration was elevated in visceral fat pads of wild-type mice consuming HFD for 20 weeks by 166±62% (p = 0.03, n≥7 per group, data not shown) compared with those from wild-type mice on LFD diet as assessed by immunhistochemical analysis of F4/80-positive cells in adipose tissue sections and real-time PCR analysis. This increase in inflammatory cell infiltration was absent in mice lacking CD40L (Figure 5 A–C). Interestingly, CD40L-deficiency did only affect total numbers of infiltrating macrophages, but not macrophage diversity, since numbers of CD11c^+^ M1-macrophages were not changed in CD40L^−/−^ mice (Figure 5 D). Also, mRNA abundance of M2-Marker Arginase-1 was not changed in adipose tissue (data not shown). B-cells infiltrate adipose tissue upon DIO as shown recently [15] and were reduced in CD40L^−/−^ mice in our study (Figure 5 D). As previously described, CD8+ T-cell content rose upon consumption of HFD [9], but was not affected by CD40L-deficiency in both groups (Figure 5 F). Likewise, number of CD4+ T-helper cells showed no differences between the groups (Figure 5 G). Regulatory T-cells – physiologically resident in lean adipose tissue [11] – increased in adipose tissue of CD40L^−/−^ mice consuming HFD compared with wild-type mice consuming HFD (Figure 5 H), suggestive of a protective phenotype in these mice. Adipocyte size correlates with obesity and the metabolic syndrome [36]. In CD40L-deficient mice mean adipocyte diameter was reduced compared with corresponding wildtype mice after HFD-feeding (Figure 5 I). Also, relative cholesterol content tended to decrease in adipocyte lysates of CD40L^−/−^ mice (Table 1).

**Figure 6:**
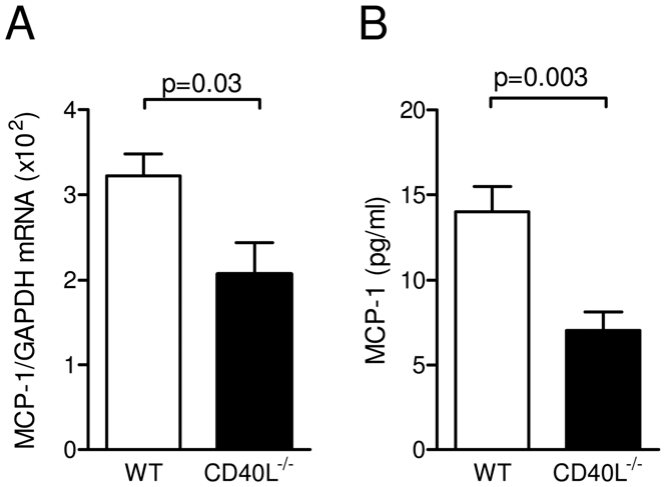
Genetic absence of CD40L reduces MCP-1 gene expression in adipose tissue and MCP-1 plasma levels. Diet-induced obesity was induced by feeding with a high fat diet for 20 weeks. MCP-1 gene transcripts in adipose tissue were quantified by quantitative PCR after whole tissue RNA preparation and normalization for GAPDH expression (A). Levels of circulating MCP-1 were determined in plasma by cytometric bead array. Data are presented as mean ± SEM of at least 6 animals per group.

To explore potential mechanisms of these findings we evaluated chemokine expression in adipose tissue. We previously reported that CD40L induces secretion of pro-inflammatory cytokines and chemokines including MCP-1 in various cell types including adipose cells [27], [37]. Whereas gene expression of both chemokines RANTES and MCP-1 were induced by HFD (data not shown), only MCP-1 proved to be regulated by CD40L showing a significant reduction in adipose tissue of CD40L^−/−^ mice consuming HFD compared with respective controls (Figure 6 A). Accordingly, CD40L-deficient mice consuming HFD for 20 weeks had significantly lower plasma levels of MCP-1 (Figure 6B), while levels of other inflammatory cytokines, such as IL-12, IL-10, IFN-γ, IL-6 or TNF-α were not modulated by CD40L-deficiency after HFD-consumption (Figure S3 A–E), indicating that CD40L specifically regulates gene expression of MCP-1.

### Deficiency of CD40L modifies balance of B-cell subsets and reduces levels of circulating auto-antibodies against oxidized lipids

**Figure 7:**
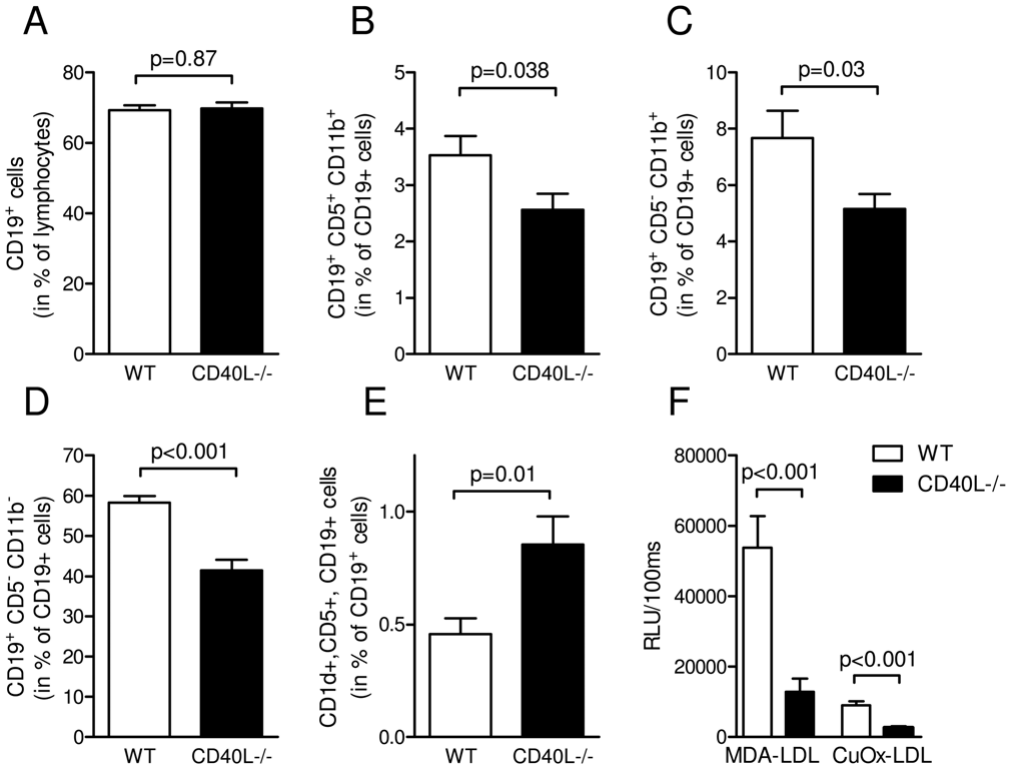
Deficiency of CD40L modifies balance of B-cell subsets and reduces levels of circulating auto-antibodies against oxidized lipids. Numbers of peripheral CD19-positive B-cells (A), peritoneal CD19^+^CD5^+^CD11b^+^ B1a-cells (B), peritoneal CD19^+^CD5^−^CD11b^+^ B1b-cells (C), and peritoneal CD19^+^CD5^−^CD11b^−^ B2-cells (D) were determined by flow cytometry. Regulatory B-cells were defined as CD19^+^CD5^+^CD1d^+^ cells in the peritoneal cavity (E). Levels of circulating antibodies against the indicated epitopes were determined by chemiluminescent ELISA (F). Data are presented as mean ± SEM of at least 15 animals per group.

CD40L-dependent co-stimulatory pathways regulate fundamental B-cell functions, such as activation, proliferation, and induction of IgG-antibody class switch [20]. Since B-cell accumulation in adipose tissue was reduced in mice lacking CD40L, we explored whether CD40L deficiency alters balance of different B-cell subsets. While total numbers of peripheral CD19^+^ B-cells were not affected by the absence of CD40L (Figure 7A), B-cells resident in the peritoneal cavity decreased in CD40L^−/−^ mice. This decrease was observed in both, innate-like B-cell subsets, such as B1a and B1b cells (Figure 7B, C), and in conventional B2-cells which mainly act in acquired immunity (Figure 7D). Surprisingly, regulatory CD1d^+^CD5^+^CD19^+^ B-cells – regarded as distinct subset suppressing T-cell mediated inflammation and thereby exhibiting anti-inflammatory actions [38] – increased by 88±27% in CD40L^−/−^ mice (p = 0.01, n≥18 per group). These data suggest a shift towards anti-inflammatory, suppressive B-cell subsets in CD40L-deficient mice. The increase in protective B-regulatory cells was also observed in spleens of CD40L^−/−^ mice (data not shown). DIO was recently reported to induce production of pathogenic, auto-reactive IgG antibodies by B2-cells [15]. Since LDL plasma levels strongly increased in DIO mice (Figure 4B) and diabetes is associated with increased oxidative stress, we tested the occurrence of OxLDL-specific antibodies in our mice. IgG-antibodies against malondialdehyde-modified LDL (MDA-LDL) and copper-oxidized LDL (CuOx-LDL) were strongly up-regulated in HFD-consuming wildtype mice compared with LFD. Interestingly, this increase in anti-OxLDL antibodies was absent in CD40L-deficient mice (Figure 7F), suggesting that the effects observed might be caused by reduced levels of pro-inflammatory IgG antibodies.

## Discussion

Chronic inflammation plays a key role in obesity, and hallmarks of adipose tissue inflammation, such as infiltration of immune cells and inflammatory cytokine production are instrumental for the pathogenesis of various other inflammatory pathologies, such as atherogenesis [4]. Despite the extensive characterization of cellular fractions resident in adipose tissue and their contribution to adipose tissue inflammation, the exact pro-inflammatory pathways underlying these phenomena remain incompletely understood. CD40L potently regulates B- and T-cell function and figures prominently in the initiation and progression of a variety of chronic inflammatory diseases, including atherosclerosis [20], [22]. Here, we report that genetic deficiency of CD40L attenuates adipose tissue inflammation in a mouse model of diet-induced obesity. Most likely, this effect was caused (1) by down-regulation of the pro-inflammatory chemokine MCP-1 resulting in reduced immune cell recruitment to inflamed adipose tissue and (2) by alteration of B-cell functioning and decreased levels of pathogenic IgG antibodies. MCP-1, a potent chemotactic factor for monocytes implicated in a variety of inflammatory diseases including atherosclerosis [39], has previously been shown to regulate inflammatory cell infiltration to adipose tissue in diet-induced obesity [40]. *In vitro*, our group and others recently demonstrated initiation of MCP-1 gene- and protein expression by CD40L in several cell types including adipose cells [27], [31], [37]. Interstingly, expression of other inflammatory chemokines such as RANTES and CXCL-1 remained unaffected by CD40L deficiency pointing towards a specific role of MCP-1 in adipose tissue inflammation. Notably, regulation of MCP-1 gene expression might not be restricted to adipocytes, but could also be secondary to diminished inflammatory cell recruitment in these mice.

Adipose tissue inflammation is orchestrated by infiltration, accumulation, and interaction of a plethora of immune cells, such as B- and T-cells, macrophages, and mast cells. Interestingly, B-cell infiltration occurs early in disease progression and seems to promote further disease progression [15], [41]. In our study, B-cell accumulation in adipose tissue was strongly reduced in CD40L^−/−^ mice, while B-cell diversity shifted towards anti-inflammatory subsets, such as B-regulatory cells. This is supported by numerous reports showing that CD40L-associated pathways fundamentally regulate B-cell physiology and pathophysiology in various disease models [20], [42]. We also propose that the production of pathogenic antibodies by B-cells, as exemplarily shown for OxLDL, contributes to the effects observed as previously suggested by others [15]. As demonstrated previously, oxidation-specific epitopes, such as oxidized LDL represent major targets of protective natural IgM antibodies [43], but are also recognized by potentially pathogenic T-cell dependent IgG antibodies as a result of pathological T-cell activation, which is impaired in CD40L deficient mice. We cannot exclude that regulation of immune cells apart from B-cells by B-regulatory cells might result in a protective phenotype. Also, infiltration of T-cell subsets, such as CD4^+^, CD8^+^, and T-regulatory cells, was identified as crucial event in the initiation of adipose tissue inflammation [9], [11], [44]. However, the content of CD8+ and CD4^+^ cells was not altered by CD40L deficiency in our study. Numbers of regulatory T-cells decreased during DIO and persisted on a higher level in CD40L^−/−^ mice. Of note, a decrease of T regulatory cells has been proposed to mediate the onset of the metabolic syndrome while accumulation of CD8+ T-cells has been suggested to promote disease progression [11]. This is well in accord with our findings that diet-induced fat depositions as assessed by MRI body composition analysis were only blunted in the early stages of diet-induced obesity by CD40L-deficiency. Also, the slight increase in relative weight gain occurred in the late stages of DIO in CD40L^−/−^ mice, while total body weight did not differ between both genotypes. In accord with this, it has been shown that both, B-cell deficiency and B-cell depletion reduce inflammatory cell accumulation in adipose tissue while not affecting the development of obesity [15]. In fact, CD40L might be more important for the initiation of obesity, rather than its propagation.

Importantly, food intake, heat production, and respiratory exchange ratio were not altered in CD40L^−/−^ mice, ruling out an effect of CD40L on basic energy metabolism affecting our results. However, we cannot exclude that CD40L directly affects the regulation of growth hormones since levels of somatostatin, GnRH or IGF-1 were modulated in CD40L^−/−^ mice on LFD, potently affecting weight and metabolic characteristics in these mice. However, growth hormone levels were not altered in mice consuming HFD.

Insulin resistance is now widely accepted as part of the metabolic syndrome. Poggi et al. demonstrated that stimulation with recombinant CD40L impaired insulin-induced glucose uptake in adipocytes by down-regulating expression of insulin receptor-1 (IRS-1) and glucose transporter-4 (GLUT-4), proposing CD40L as direct regulator of insulin and glucose tolerance [31]. In accord with these findings, we observed increased IRS-1 and GLUT-4 mRNA expression in liver and skeletal muscle. Also, insulin levels were strongly down regulated in CD40L-deficient mice under both dietary regimens, suggesting a role of CD40L in insulin resistance. However, we could not confirm functional relevance of these observations in CD40L^−/−^ mice, since fasting glucose levels and GTT/ITT did not differ between CD40L^−/−^ and corresponding WT mice consuming HFD for 20 weeks. On the other hand, CD40L-deficient mice consuming LFD demonstrated improved GTT and ITT (data not shown), but these effects might be secondary to diminished weight gain and smaller fat depots in these mice [45]. Conversely, in clamps analysis, GIR tended to decrease in CD40L^−/−^ mice on HFD, which might be caused by diminished glucose uptake in skeletal muscle – but not in visceral adipose tissue – as observed in our study. These findings indicate a trend towards an aggravation of muscle insulin sensitivity in CD40L^−/−^ mice – albeit without systemic relevance. Overall, more mechanistic workup, including description of insulin secretion and insulin receptor signaling in CD40L-deficient mice will be needed to define the exact role of CD40L in insulin and glucose homeostasis.

Obesity correlates with hepatic steatosis [46]. Surprisingly, we found that mice lacking CD40L exhibited a strong increase in hepatic accumulation of triglycerides and cholesterol. Histological analysis showed augmented vacuole formation and an increased number of lipid droplets, consistent with aggravated hepatic steatosis. In line with our findings, Villeneuve et al. recently proposed CD40L as protective factor of hepatic steatosis demonstrating increased hepatic steatosis in CD40L-deficient mice consuming a diet rich in olive oil [34]. The authors showed that this effect was most likely caused by an inhibition of apoB100 and very low density lipoprotein (VLDL) secretion in CD40L^−/−^ mice and subsequently lipid accumulation in the liver. Accordingly, we found that concentration of apoB100 containing low density lipoproteins (LDL) was markedly reduced in CD40L-deficient mice in our study, while – as previously described – total plasma cholesterol and triglycerides were not affected [47]. Since this effect was present in both diet arms of our study, we assume that CD40L might indeed be a regulator of hepatic steatosis independently of dietary free fatty acid intake. This was further supported by the fact that CD40L deficiency modulated liver-specific gene expression of transcription factors and enzymes linked to lipid metabolism, including PPARα, SREBP1c, or FAS. Of note, we did not observe down-regulation of apoB100 as described previously. Therefore, the exact pathways involved have to be further evaluated.

Hypertension is considered an integral part and trigger of the metabolic syndrome. Of note, systolic blood pressure in CD40L^−/−^ mice was significantly higher than in corresponding WT mice at the begin of the feeding study. This increase in blood pressure was most likely not caused by a pro-inflammatory vascular phenotype. This was further supported by the fact that both, systolic blood pressure and levels of pro-inflammatory cytokines showed no difference in both dietary arms at the end of the study.

Since reduction of inflammatory cell accumulation in adipose tissue occurred in CD40L-deficient mice without affecting the metabolic phenotype, our findings furthermore propose the existence of specific inflammatory pathways in DIO. In line with this, the inflammatory stimulus of HFD might overwhelm the anti-inflammatory capacity in CD40L-deficient animals. This might also be reflected by the fact that inflammatory cytokine levels were blunted in CD40L^−/−^ mice on LFD, but not on HFD.

In summary, we present the novel finding that deficiency of CD40L specifically attenuates diet-induced adipose tissue inflammation in mice while not affecting DIO itself and its associated metabolic complications, such as insulin resistance. Our data contribute to the understanding of inflammatory pathways in the metabolic syndrome and may have implications on the development of novel anti-inflammatory therapeutic strategies to fight MS and other chronic inflammatory conditions.

## Materials and Methods

### Ethics statement

All experimental protocols were approved by the animal ethics committee (AEC) of the Alfred Medical Research and Education Precinct (AMREP), Melbourne, Australia under the AEC approved project number E/0779/2008/B.

### Animals

CD40L^−/−^ mice on a C57BL/6J background and aged-matched wildtype mice (WT) were obtained from Jackson Laboratory (USA). To induce the metabolic syndrome 8-week old, male animals received a high-fat diet (HFD) containing 23% fat, 18.4% protein, 16.8 MJ/kg digestible energy (Research Diet D12451, Specialty Feeds, Australia) for 20 weeks. Control animals consumed LFD containing 4.6% fat, 19.6% protein, 14.3 MJ/kg digestible energy (Specialty Feeds, Australia). All mice were maintained under a 12-hour light, 12-hour dark cycle and had access to food and water ad libitum unless indicated otherwise. At the end of the study overnight-fasted animals were euthanized, blood and organs were collected and analyzed as described below.

### Body composition analysis

Composition of total fat, lean and water mass was assessed by MRI-based body composition analysis (EchoMRI, Echo Medical Systems, USA) on a basis of 4-weekly follow-up measurements.

### Intraperitoneal glucose and insulin tolerance testing

Glucose and insulin tolerance testing was performed after 8 and 20 weeks after start of the diet. For glucose tolerance tests (GTT) mice were deprived of food 6 hours prior to the procedures and received intraperitoneal glucose injections (1 g/kg lean body mass as assessed by body composition analysis). For insulin tolerance tests (ITT) mice were injected with human insulin intraperitoneally (0.5 U/kg, Actrapid Insulin, Novo Nordisk, Denmark). Blood samples were collected at 0, 15, 30, 45, 60, 90 and 120 minutes after the insulin challenge. Plasma glucose concentration was measured with AccuChek GO (Roche Diagnostics, Switzerland).

### Insulin measurements

Plasma insulin levels were determined at the end of the feeding study by a commercial ELISA kit (Millipore). Animals were deprived of food 6 hours prior to blood sampling.

### Euglycemic-hyperinsulinemic clamp (EHC)

After 20 weeks on diet animals were subjected to euglycemic-hyperinsulinemic clamps. Four days prior to the euglycemic-hyperinsulinemic clamp mice were anaesthetized and an indwelling catheter was placed into the right internal jugular vein. Mice were allowed to recover for 4 days following surgery and body weight was monitored daily. On the day of the clamp mice were fasted for 5 hours followed by a continuous infusion of [3-^3^] Hg-labeled glucose (5 µCi bolus and 0.05 µCi/min) to measure basal glucose turnover. After infusion of the tracer for 2 hours EHC was performed. A continuous infusion of insulin (4 mU/kg/min) was started. Blood (5 µL) was sampled from the tail vein every 10 min to measure glucose levels and euglycemia was maintained using a variable infusion of 30% glucose. To determine glucose specific activity, small blood samples were taken regularly during the clamp. To measure the tissue glucose uptake, a bolus of 2[^14^C] deoxyglucose (12 µ Ci) was injected. Blood concentrations of [3-^3^H] glucose and 2[^14^C] deoxyglucose were determined after deproteinization using BaOH and ZnSO_4_. Glucose uptake into individual tissues was assessed by determining the tissue content of 2[^14^C] deoxyglucose-6-phosphate and the plasma 2 [^14^C] deoxyglucose profile.

### Metabolic caging

Animals were allowed to acclimatize to metabolic cages (Oxymax/CLAMS, Columbus Instruments, USA) for a period of 24 hours before start of analysis. During the 24 hour observation period food intake, ambulatory movement, heat production, O_2_ consumption and CO_2_ production were assessed and recorded.

### Preparation of the stromal vascular fraction

Murine epididymal fat pads were minced and digested with collagenase for 30 minutes at 37°C (0.5 mg/ml, Collagenase B, Roche, Switzerland). Digested tissue was filtered through a nylon mesh (100 µm). The stromal vascular fraction (SVF) and adipocyte fraction were obtained from the resulting pellet and supernatant, respectively.

### Preparation of splenocytes

Cells were released from spleen by nicking the capsule and gently rotating two microscope slides. Cell suspension was filtered through a nylon mesh (100 µm) and pelleted.

### Flow cytometry

The stromal vascular fraction (SVF) and splenocytes were obtained as described above. Remaining red blood cells were removed by FACS Lysing solution (BD Biosciences, USA). Cells were washed in PBS and Fc-Receptors were blocked by anti-CD16/CD32 (eBioscience, USA) for 10 minutes on ice. Cells were then labeled with the indicated antibodies before quantification with a flow cytometer (BD FACS Canto II, BD, USA). Following antibodies were used: anit-CD8-PE, anti-CD4-FITC, anti-CD3-APC, anti-CD25-PE, anti-Foxp3-APC, anti-CD19-APC, anti-CD115-PE, anti-Ly6G/C-FITC, anti-F4/80-PE and anti-CD11c-APC (all from eBioscience, USA).

### Isolation of total RNA and quantitative real-time PCR

Organs were stored in RNAlater (Qiagen, USA) after harvest. RNA was extracted from epididymal adipose tissue using TRIzol Reagent (Invitrogen, USA) and glycogen as a coprecipitator (Roche, Switzerland). Homogenization was performed using a rotor-stator dispergator (IKA, Germany). 1 µg of total RNA was transcribed into cDNA using the Transcriptor First Strand cDNA Synthesis Kit (Roche, Switzerland). Subsequent quantitative real-time PCR was performed with a LightCycler 480 System with the LightCycler 480 SYBR Green I Master (Roche, Switzerland) detection format. Murine GAPDH served as control. Primer sequences are avaible upon request.

### Histology, immunhistochemistry (IHC)

Adipose tissue was fixed for 24–48 hours with 10% paraformaldehyde solution (Sigma-Aldrich, USA) at 4°C, dehydrated, embedded in paraffin, and cut into 6 µm sections. Sections were mounted on glass slides and depleted of paraffin and rehydrated. Sections were then placed in pre-heated Target Retrieval Solution (Dako, USA) and rinsed with PBS. For IHC staining sections were blocked with 4% rabbit serum, incubated for 1.5 hours at RT with anti-F4/80 (Abcam, USA) and detected by an appropriate secondary antibody followed by detection with AEC+ substrate (DAKO, USA). Liver sections were fixed in 10% neutral buffered formalin (Harleco, USA) for 10 minutes, rinsed with water, submerged in 100% Polypropylene glycol (Fischer Chemicals, Switzerland) for 2 minutes, incubated in Oil-Red-O staining solution (Sigma-Aldrich, USA) for 25 minutes at 60°C, washed in Millipore water and 0.05% Ammonia H_2_O.

### Analysis of murine plasma samples

Plasma levels of insulin, leptin, adiponectin, FFAs and the indicated growth hormones were measured by ELISA according to the manufacturers' protocols (Mercodia, Sweden; R&D Systems, USA; Cusabio, USA; Uscn Life Science Inc, China). Triglyceride, LDL, HDL, and total cholesterol levels of murine plasma samples were assessed by COBAS Integra 400 plus system (Roche Diagnostics, Switzerland). Plasma cytokines were determined by a cytometric bead array (CBA, BD Biosciences, USA) according to the manufacturers' protocol.

### Liver Homogenisates

Liver samples were homogenized in 200 µl PBS using a dispergator (IKA, Germany) and normalized to a protein concentration of 2.5 mg/ml. Lipids were extracted according to Folch et al. [48]. Samples were analyzed by liquid chromatography-mass spectrometry on a 4000QTRAP™ LC/MS/MS system (Applied Biosystems, USA).

### Measurement of antibody titers

Plasma titers of antibodies to the indicated antigens were determined by chemiluminescent ELISA as previously described [49].

### Statistical analysis

Data are presented as means ± SEM and were analyzed by 2-tailed Student's t-test. P-values<0.05 were considered significant.

## Supporting Information

Figure S1 **WT and CD40L^−/−^ mice consumed a standard low fat diet (LFD) for 20 weeks.** Relative increase of body weight (shown as % of body weight at week o) and total body fat as assessed by MRI-based body composition analysis are shown for the indicated time points (A, B). Weight of epididymidal (EFP), peri-renal (RFP), and cardiac fat pads (CFP) fat are displayed as percentage of total body weight (BW, C). Plasma levels of leptin were determined at the indicated time points (D). Food intake (E), heat production (F), ambulatory movement (G), and respiratory exchange ratio (RER, H) were analyzed in metabolic cages. Growth hormone levels were determined by ELISA (I–K). Data are presented as mean ± SEM of at least 15 mice per group (A–D, H–J) or 9 mice per group (E–H).
(TIF)

Figure S2 **Hyperinsulinemic-euglycemic clamp analysis after 20 weeks of HFD revealed rate of glucose infusion rate (GIR, A), glucose disappearance under clamp (Ins-GDR, B) and basal conditions (GDR, C).** Glucose uptake in peripheral muscle and heart tissue (D) and fat tissue (E) was determined by specific glucose tracer uptake. RNA abundance of insulin-receptor 1 (IRS-1), glucose transporter 4 (GLUT-4) and phosphoenolpyruvate carboxykinase (PEPCK) was quantified by real time PCR and normalization to GAPDH (F–K). Data are presented as mean ± SEM of at least 9 mice per group.
(TIF)

Figure S3 **Plasma levels of inflammatory cytokines were determined by cytometric bead array after 20 weeks of standard diet (LFD) or high fat diet (HFD).**
(TIF)
