# Correction: CD40L Deficiency Attenuates Diet-Induced Adipose Tissue Inflammation by Impairing Immune Cell Accumulation and Production of Pathogenic IgG-Antibodies

**DOI:** 10.1371/annotation/84102aae-770a-41ae-9c15-4736ed129c17

**Published:** 2012-05-29

**Authors:** Dennis Wolf, Felix Jehle, Alexandra Ortiz Rodriguez, Bianca Dufner, Natalie Hoppe, Christian Colberg, Andrey Lozhkin, Nicole Bassler, Benjamin Rupprecht, Ansgar Wiedemann, Ingo Hilgendorf, Peter Stachon, Florian Willecke, Mark Febbraio, Christoph J. Binder, Christoph Bode, Andreas Zirlik, Karlheinz Peter

Constantin von zur Muhlen was inadvertently excluded from the by-line of the published article. Dr. von zur Muhlen should be listed as the 15th author and is affiliated with: 1 Atherogenesis Research Group, Department of Cardiology, University of Freiburg, Freiburg, Germany. The author's contributions are: Analyzed the data, Contributed reagents/materials/analysis tools, wrote the manuscript. 

