# Supplementary figures and images for: CD40L Deficiency Attenuates Diet-Induced Adipose Tissue Inflammation by Impairing Immune Cell Accumulation and Production of Pathogenic IgG-Antibodies
Source: PLoS One. 2012 Mar 8;7(3):e33026. doi: 10.1371/journal.pone.0033026 (PMC3297623; doi:10.1371/journal.pone.0033026)

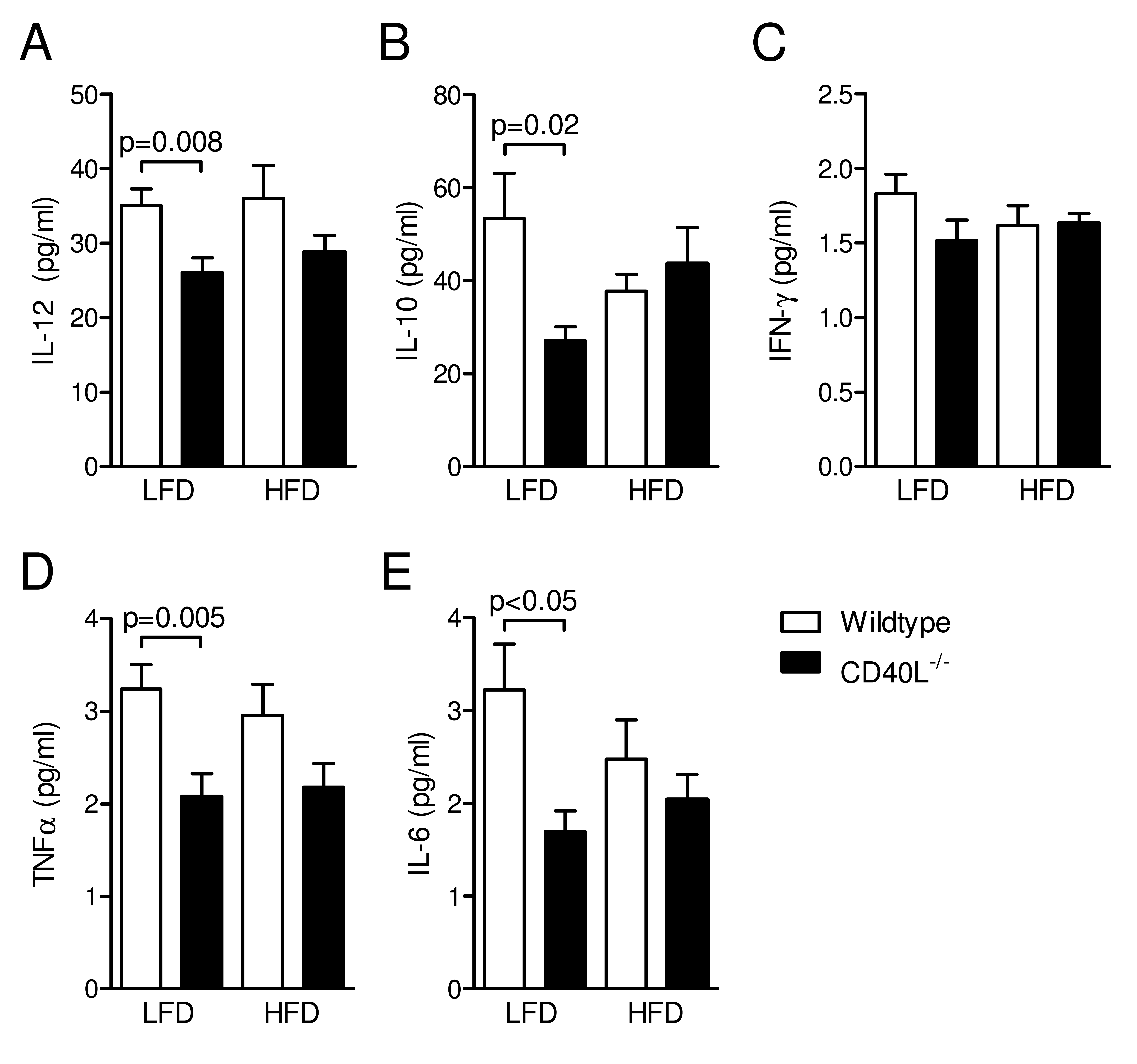

Supplement: Figure S3 — Plasma levels of inflammatory cytokines were determined by cytometric bead array after 20 weeks of standard diet (LFD) or high fat diet (HFD). (TIF) [file pone.0033026.s003.tif]
